# Multidisciplinary approach to the accurate diagnosis of parosteal osteosarcoma

**DOI:** 10.1016/j.radcr.2025.04.010

**Published:** 2025-04-29

**Authors:** Miroslav Kilian, Radovan Vanatka, Radka Tomasova, Iveta Meciarova, Peter Hlavcak, Radoslav Zamborsky, Silvia Vajczikova, Miroslav Tomka

**Affiliations:** a Second Orthopedic and Traumatology Department, University Hospital Bratislava and Comenius University, Bratislava, Slovak Republic; b Trauma Surgery Clinic, University Hospital Bratislava and Slovak Medical University, Bratislava, Slovak Republic; c Department of Radiology, University Hospital Bratislava and Comenius University, Bratislava, Slovak Republic; d Department of Genetics, Medirex Ltd., Bratislava, Slovak Republic; e Unilabs Slovakia Ltd., Diagnostic Center of Pathology Bratislava, Slovak Republic; f Department of Orthopaedics, Comenius University Bratislava and the National Institute of Children’s Diseases, Bratislava, Slovak Republic; g Faculty of Medicine, Institute of Medical Biology, Genetics and Clinical Genetics, Comenius University, Bratislava, Slovak Republic

**Keywords:** Parosteal osteosarcoma, Osteosarcomas, Myositis ossificans, Delayed diagnosis of parosteal osteosarcoma

## Abstract

The successful treatment of any disease depends on early and correct diagnosis. A failure to establish an accurate diagnosis can lead to ineffective, often expensive treatment and risky progression of the disease with all the negative consequences, including incurability and death. The parosteal osteosarcoma is an illustrative example of how determining the correct diagnosis can be a challenging task. Parosteal osteosarcoma is a rare malignant bone tumor, mainly affected metaphysis of long bones. It arises from cortical surface of bone and it is usually slowly growing, low-grade, and well-differentiated tumor with good prognosis. Unfortunately, parosteal osteosarcoma is often misdiagnosed as a benign osteochondroma or myositis ossificans what leads to ineffective treatment, disease progression and systemic spreading with later treatment complications. In this article, we discuss a case of 48-years-old male with prolonged diagnosis of parosteal osteosarcoma, initially misdiagnosed as myositis ossificans. The diagnosis was revised and corrected based on results obtained by various approaches, including radiography, ultrasound-guided biopsy, histology, conventional cytology, and molecular biology. On this example, we demonstrate the importance of interdisciplinary collaboration between experts from different areas of health care.

## Introduction

Osteosarcomas (OS) are primary malignant bone tumors. They arise as a product of tumor cells which produce bone and osteoid tissue. OS constitute a group of lesions characterized by distinct clinical and pathological features. Central (medullary) and surface (peripheral) osteosarcomas, growing from the bone center or peripheral bone surface, respectively, are well-separated entities. However, within each of these groups are recognized number of different subtypes [1].

Parosteal osteosarcoma (POS), or juxtacortical osteosarcoma, belongs to the group of surface osteosarcoma together with periosteal osteosarcoma and high-grade surface osteosarcoma. It is a well-differentiated (low-grade), slow-growing and late-metastasizing bone tumor that arise from the periosseous tissues adjacent to the cortex [2,3]. POS is the most common bone-surface tumor comprising for about 65% of juxtacortical osteosarcomas and 4% of all OS [4].

POS mainly affects long bones, most frequently posterior and distal part of the femoral metaphysis, then proximal tibial metaphysis and proximal humeral metaphysis. These 3 locations account for more than 80% lesions [4,5]. Some rare locations have also been reported, including skull [6], orbit [7], maxilla [8], mandible [9], rib [10], thumb [11], scapula [12] and some others.

While conventional osteosarcoma occurs in younger age, POS most frequently occurs in the third to fourth decade of life. However, the exact period is variable in most series [5]. POS show a mild female predominance [4,13]. It has better prognosis than other variants of osteosarcomas. Overall survival was 92% at 5 years and 87.8% at 10 years. Female gender and young age are considered to be good prognostic factors [14].

POS is cytogenetically characterized by presence of supernumerary ring chromosomes. It was shown that material of the ring chromosomes originates from chromosome 12, with 12q13-q15 as the minimal common region. This finding is characteristic for POS but is not common in conventional osteosarcomas which makes this region a specific hallmark of the POS [15,16]. In this region are localized 2 genes, *MDM2* and *CDK4* which are often amplified in low-grade osteosarcomas and their proteins are found to be overexpressed. Contrary, *MDM2/CDK4* expression was reported rarely in conventional osteosarcoma and in primary and recurrent periosteal osteosarcomas [17].

In this article, we present patient with POS who was initially misdiagnosed as myositis ossificans (MO). This delay led to the ineffective treatment and disease progression. Later, when disease was highly developed, genetic testing using conventional cytogenetics, array-based comparative genomic hybridization (aCGH) and FISH analysis identified aberration on chromosome 12 and thus provided valuable information for correction of diagnosis to POS.

## Case report

A 48-year-old male presented to our department of orthopedics and traumatology in January 2014 with tremendous swelling in the area of his left foreleg. Based on medical history, he underwent varicose vein stripping in 2005 and knee arthroscopy due to meniscal tear in 2007 (he fell off the bike). Other medical history was of no significance.

In January 2011, he presented painless swelling in the lower part of the left leg. The circumference of its proximal part was larger by 2 cm compared to the right leg. No other systemic symptoms were observed. Basic laboratory and sonographic tests were negative, radiographs were not performed. No specific treatment was initiated.

**Fig. 1:**
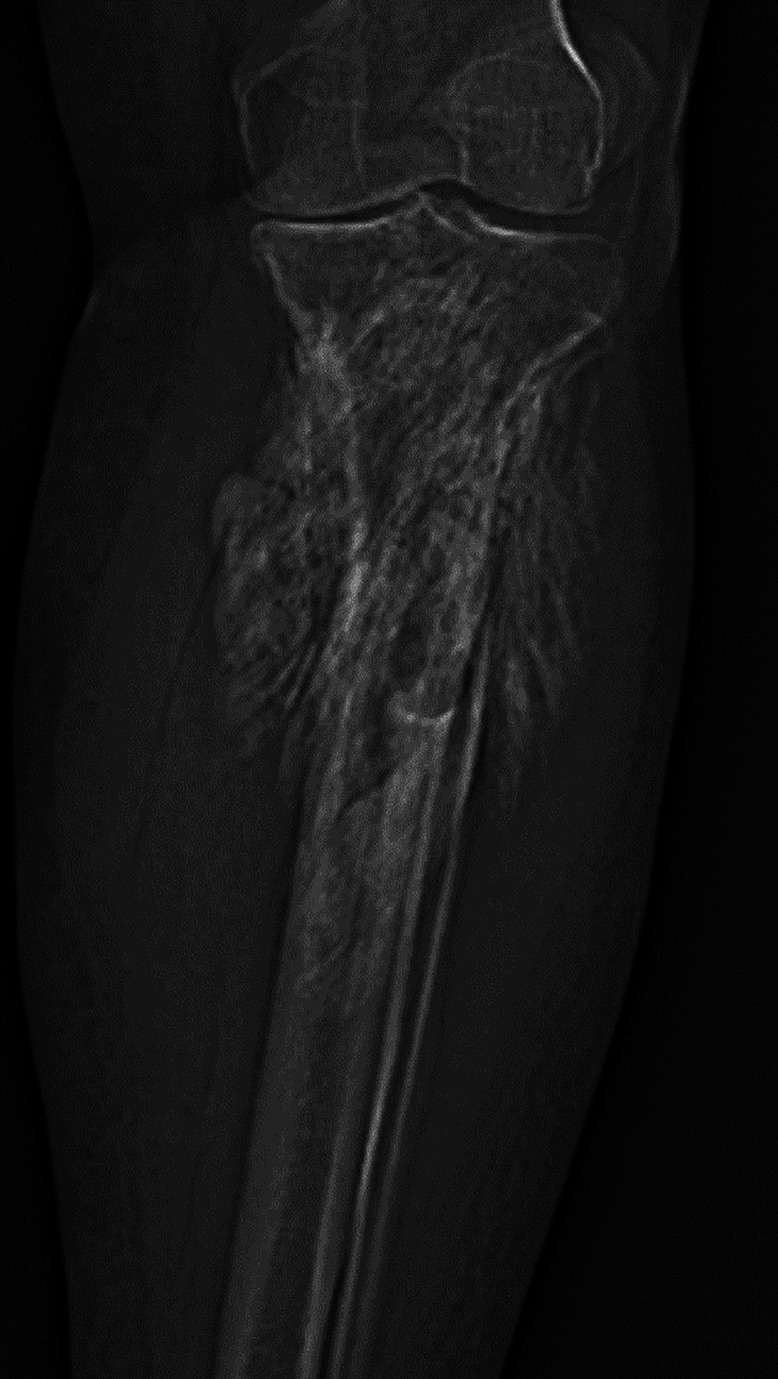
Initial radiograph of the left calf (frontal view) showing dense, inhomogenous, lobulated mass in the proximal tibial region.

**Fig. 2:**
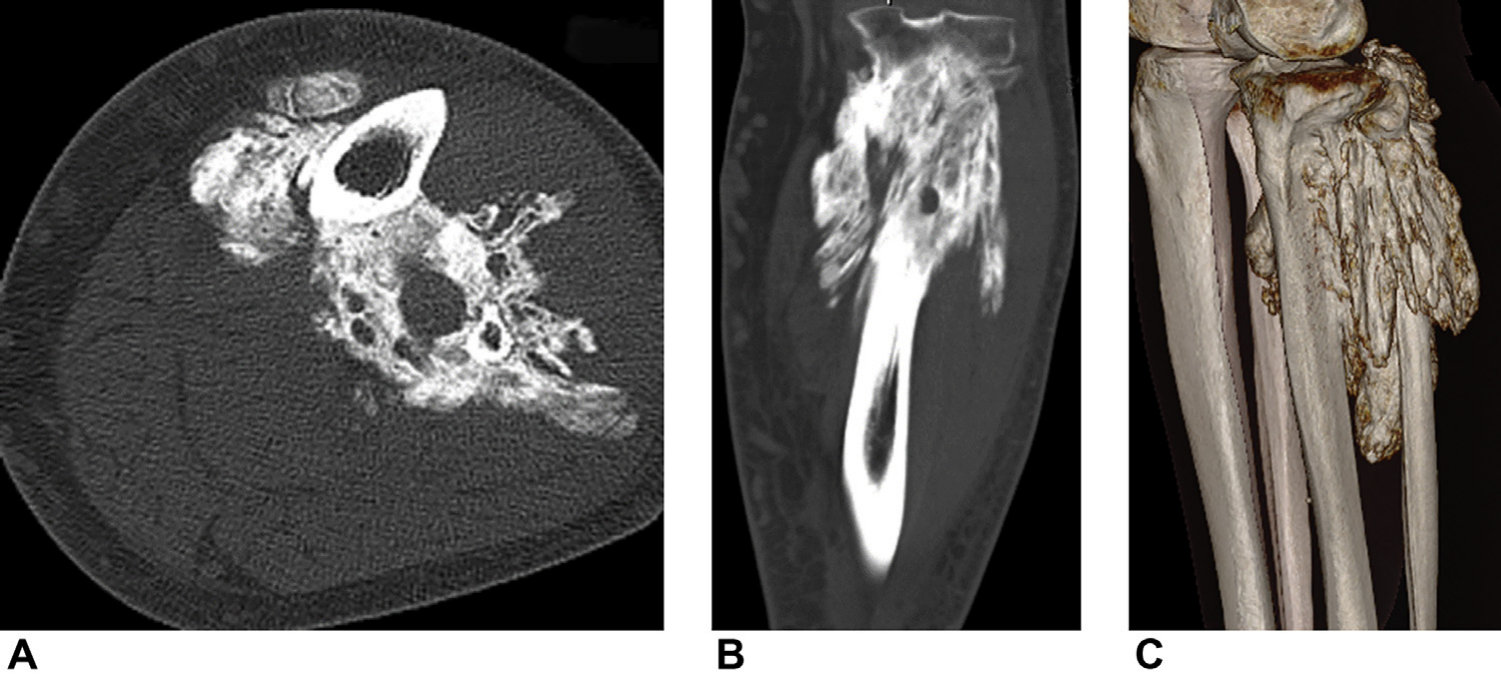
Initial CT examination (A) axial, (B) coronal, and (C) VRT image showing a well-defined, lobulated, mixed density lesion (ossified with several radiolucent soft-tissue areas) attached to the proximal part of the tibia and encircling fibula and popliteal artery and vein.

In January 2013, the patient was examined by vascular surgeon to rule out deep vein thrombosis. He complained of progressive swelling of the left ankle and foot and moderate calf pain. Sonographic Doppler tests were negative. Plain radiographs, however, showed dense, inhomogeneous, lobulated mass in the proximal tibial region (Fig. 1). The patient was referred to an orthopedic surgeon. On physical examination, solid mass was palpated in the upper third of the lower leg, mainly of its lateral aspect. Lower leg circumference of the proximal part was plus 6 cm compared to the right leg. The function of the knee and ankle joints was impaired. Computed tomography (CT) scans confirmed well-defined, lobulated, mixed density lesion attached to the proximal part of the tibia and encircling fibula and popliteal vessels, producing venostasis, edema, and dilatation of superficial veins (Fig. 2). Doppler examination confirmed compression of the popliteal vein by this mass and deep venous thrombosis. Bone scintigraphy showed inhomogeneous uptake of radioisotope by the mass that pointed to the ongoing process of ossification. The patient was then referred to another institution to undergo a surgical biopsy due to the lack of typical imaging features and possibly rule out a neoplastic process.

Four samples were taken from the anterior and anterolateral part of the tibia, fibula, and surrounding muscle tissue. The biopsy specimens were reviewed by 2 pathologists.

Based on the histopathological examination, myositis ossificans was suspected without any signs of malignancy. Due to significant expansion of ossification, amputation was recommended to the patient. He refused and sought advice from our institute.

**Fig. 3:**
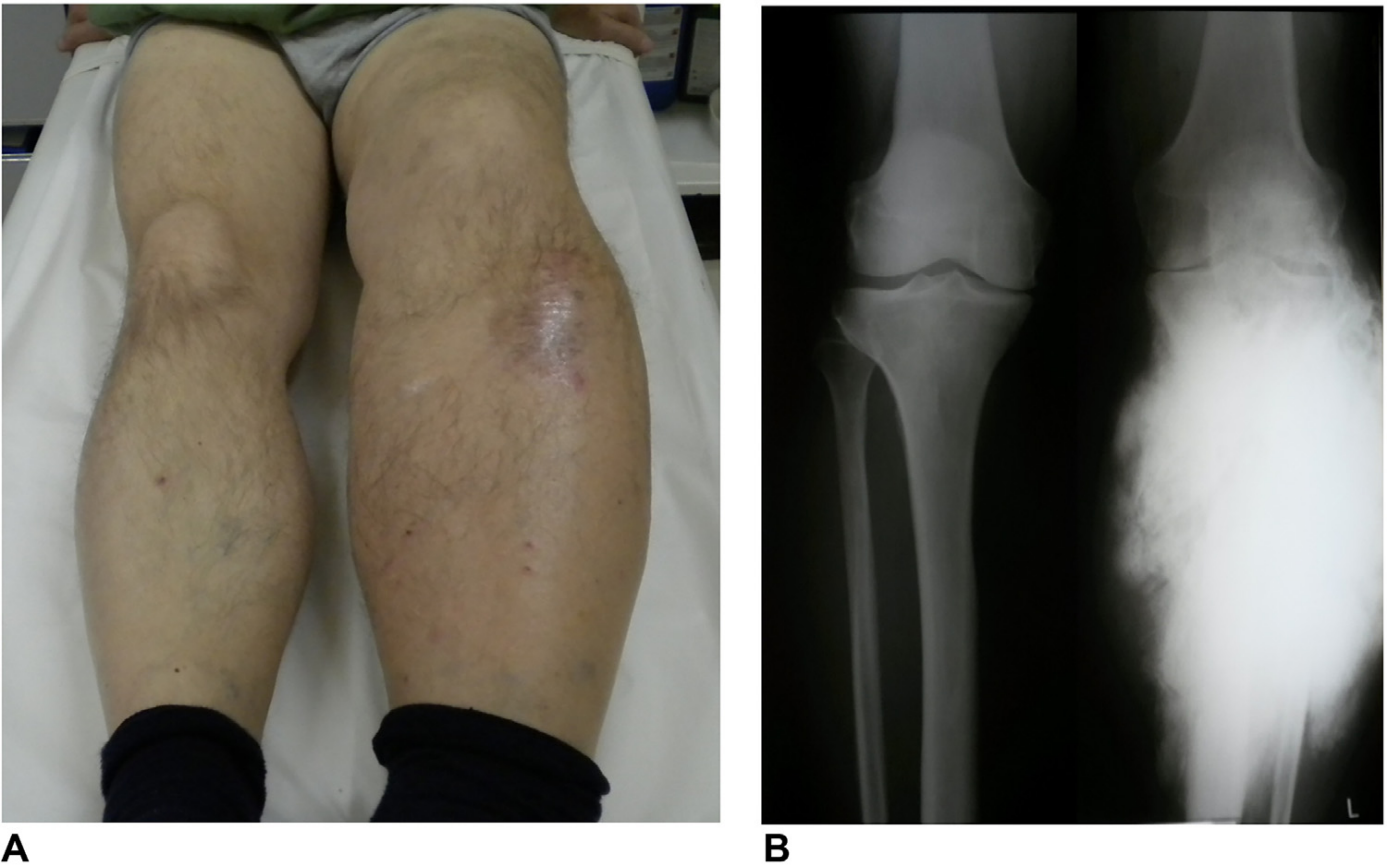
Photograph of the lower limb at the time of presentation to our institute (A). Leg circumference of the left proximal part was plus 10 cm compared to the right leg. Plain radiograph shows clear progression of the calf calcification (B).

At the time of the patient’s presentation at our institute, the circumference of the proximal part of his lower leg was larger by 10 cm compared to the right leg (Fig. 3A). Knee range of motion was 40 degrees and ankle joint function was also severely impaired. Plain radiographs showed a clear progression of the calf calcification (Fig. 3B).

**Fig. 4:**
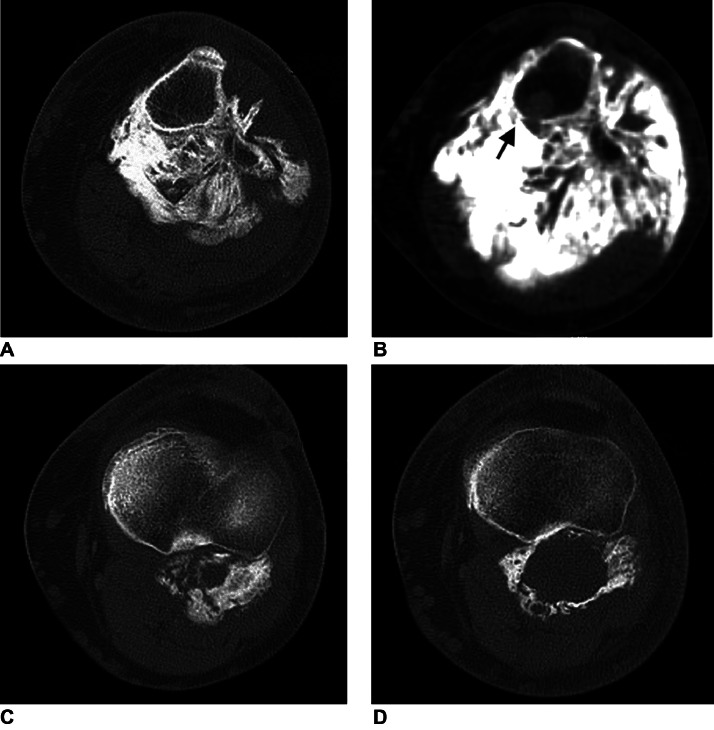
Comparison of initial (A, C) and subsequent (B, D) CT axial scans. Images show progression of size of the mass and adjacent soft-tissue areas and signs of invasion to bone marrow of tibial metaphysis (arrow).

**Fig. 5:**
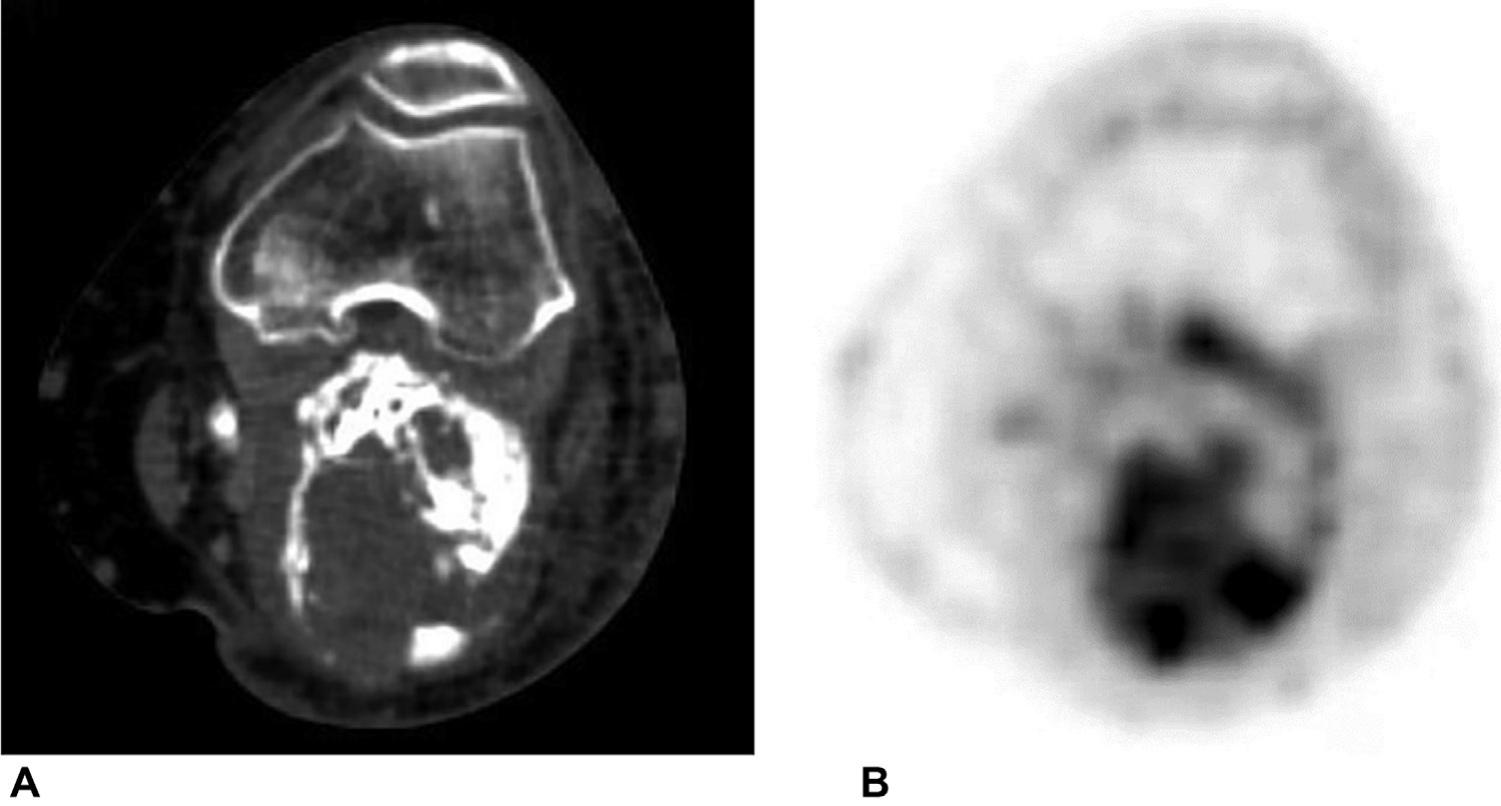
FDG PET/CT scan showing inhomogenous uptake of radioisotope predominantly in the soft-tissue areas of the mass.

**Fig. 6:**
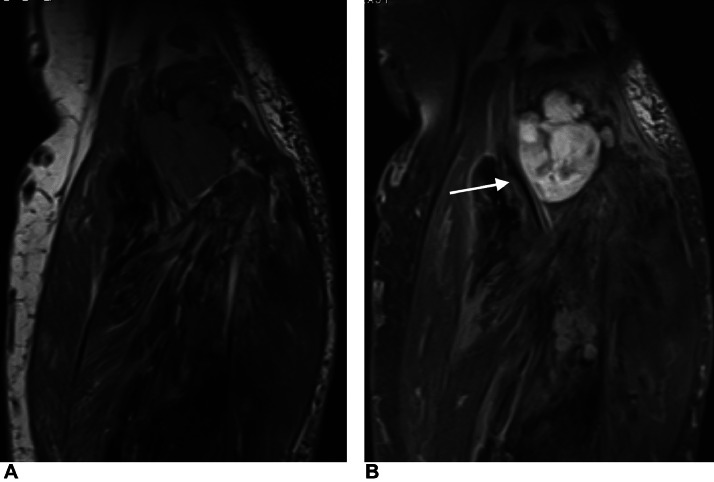
MRI image, coronal T1 tse (A), T1 tse FS (B) after gadolinium administration. Images show various levels of the contrast medium uptake in the hypointense, well-demarcated soft-tissue areas of the mass (arrow).

Subsequently, CT confirmed the calcification of the mass in the calf itself as well as the overgrowth of the surrounding soft-tissue area and signs of its invasion to bone marrow of the tibial metaphysis (Fig. 4). FDG PET/CT scans of the left calf showed inhomogeneous uptake of the radioisotope, predominantly in the soft-tissue areas of the mass (Fig. 5). Magnetic resonance image findings demonstrated well-demarcated, T1W hypointense soft tissue areas in the mass with various levels of contrast medium uptake (Fig. 6).

**Fig. 7:**
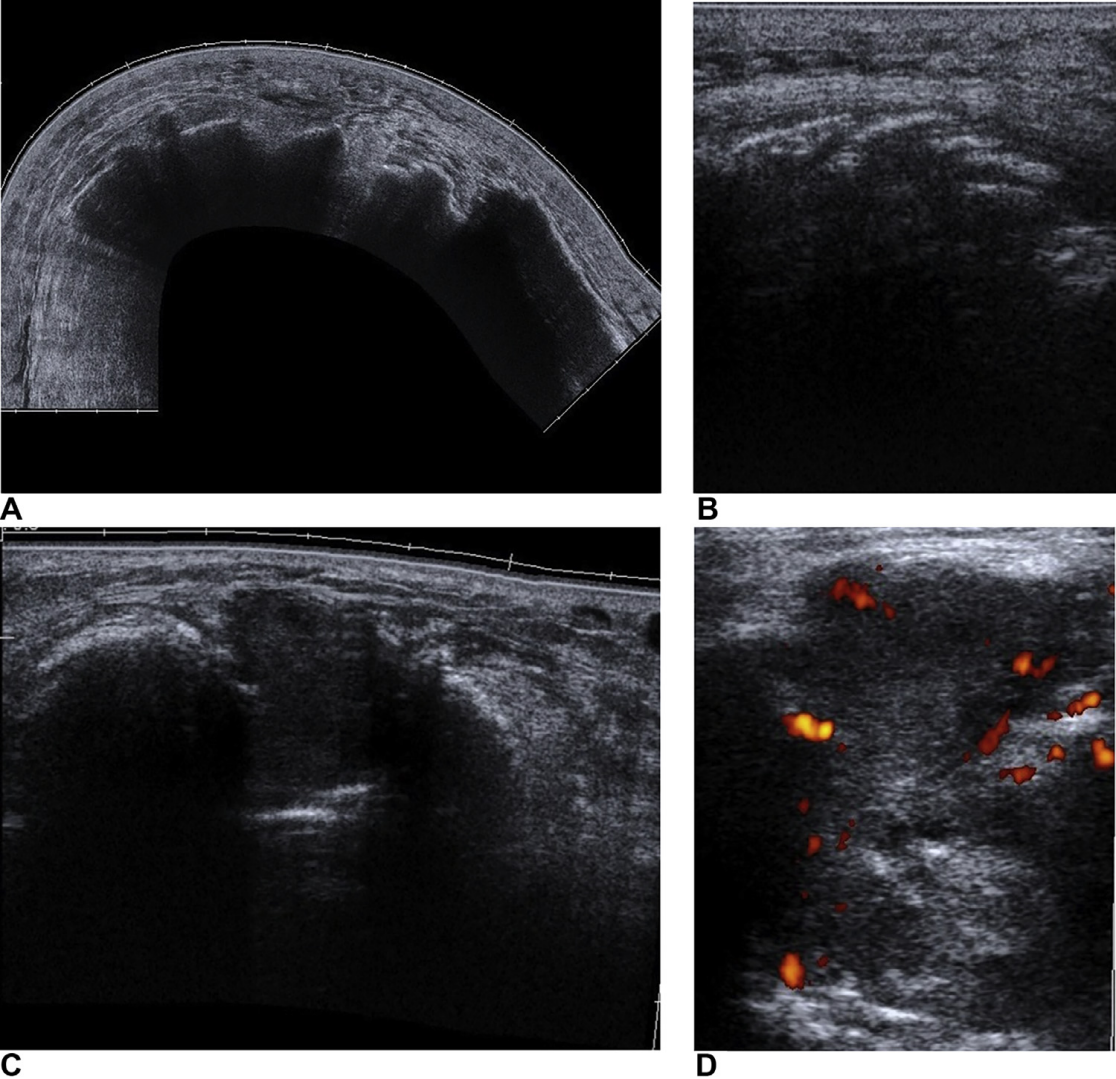
Follow-up ultrasonography showing well-demarcated, calcified mass with acoustic shadowing in the central part of left calf (A), spreading to the adjacent muscle along the muscle fascicle (B), almost anechoic soft-tissue area in the superficial part of the mass (C), distinctly vascularized (D), accessible to biopsy.

The following ultrasonography visualized 1 superficially localized soft tissue area of the mass that was almost an echogenic and vascularized (Fig. 7). It was considered to be accessible to a core-cut biopsy that was, indeed, successfully executed by guided sonography.

**Fig. 8:**
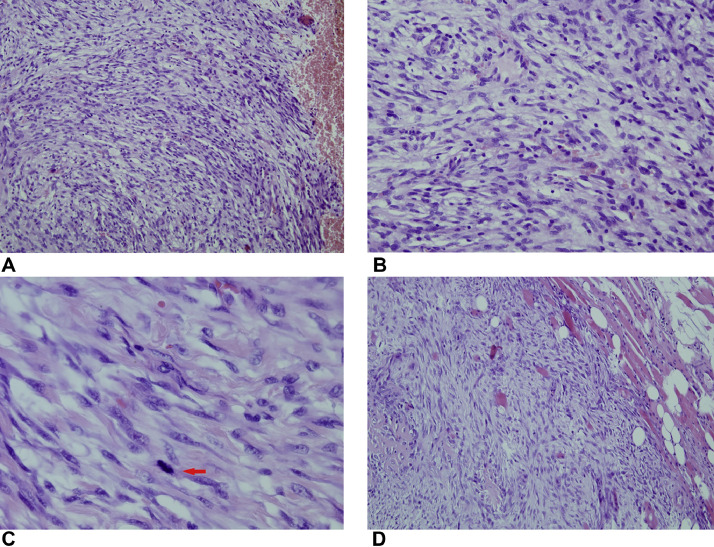
Core-cut biopsy (year 2014, A, B). Hyper-cellular, predominantly spindle cell proliferation, neoplastic cells demonstrate relatively marked pleomorphism, nuclei are spindled, fusiform, sometimes oval, unclear cytoplasmic contours, fascicular, storiform arrangement of cells, (A) H&E, x100, (B) H&E, x200. Definitive biopsy (year 2015, C, D), higher magnification, the same pattern as a year ago, cellular, predominantly spindle cell proliferation with nuclear pleomorphism, coarse chromatin, visible nucleoli, increased mitotic activity (arrow), (C) H&E, x400. (D) H&E, x100, pleomorphic spindle and oval cell proliferation with infiltrative growing into adjacent soft tissue.

The morphological pattern was completely different from the previous histological findings. The tumor was composed of hypercellular, predominantly spindle cell proliferated, neoplastic cells with relatively significant pleomorphism. The nuclei were spindled, fusiform, sometimes oval, the cytoplasmic contours were unclear (Figs. 8A and B).

**Fig. 9:**
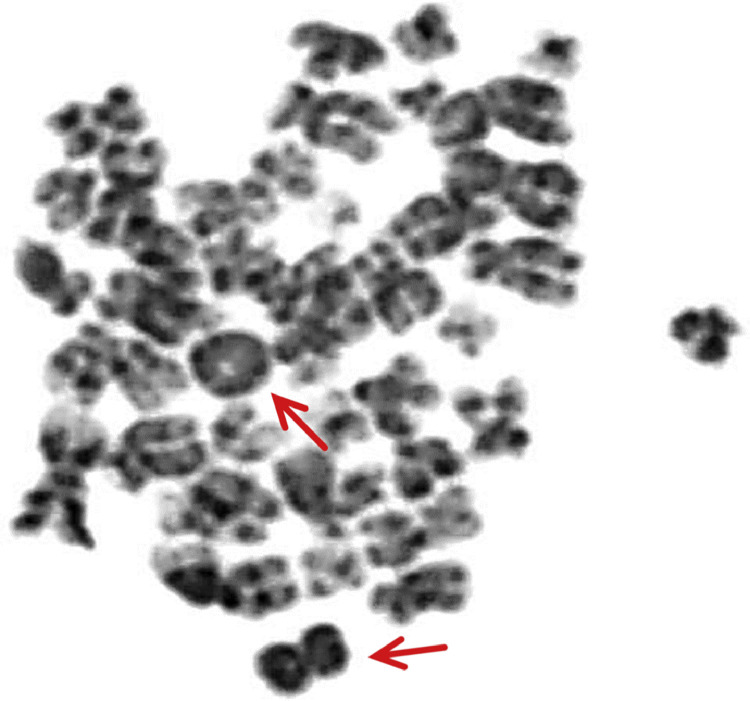
Metaphase spread showing ring chromosomes (arrows).

**Fig. 10:**
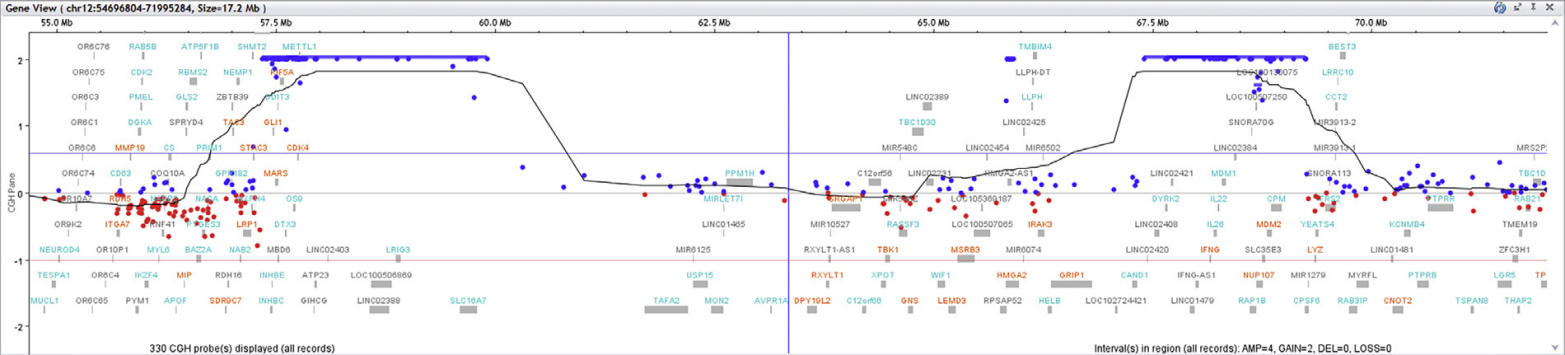
Result of array-based comparative genomic hybridization (aCGH). Blue bars indicate amplification of the genetic material from the chromosome 12 containing *CDK4* gene (12q13.3-q14.1) and *MDM2* gene (12q15), respectively.

**Fig. 11:**
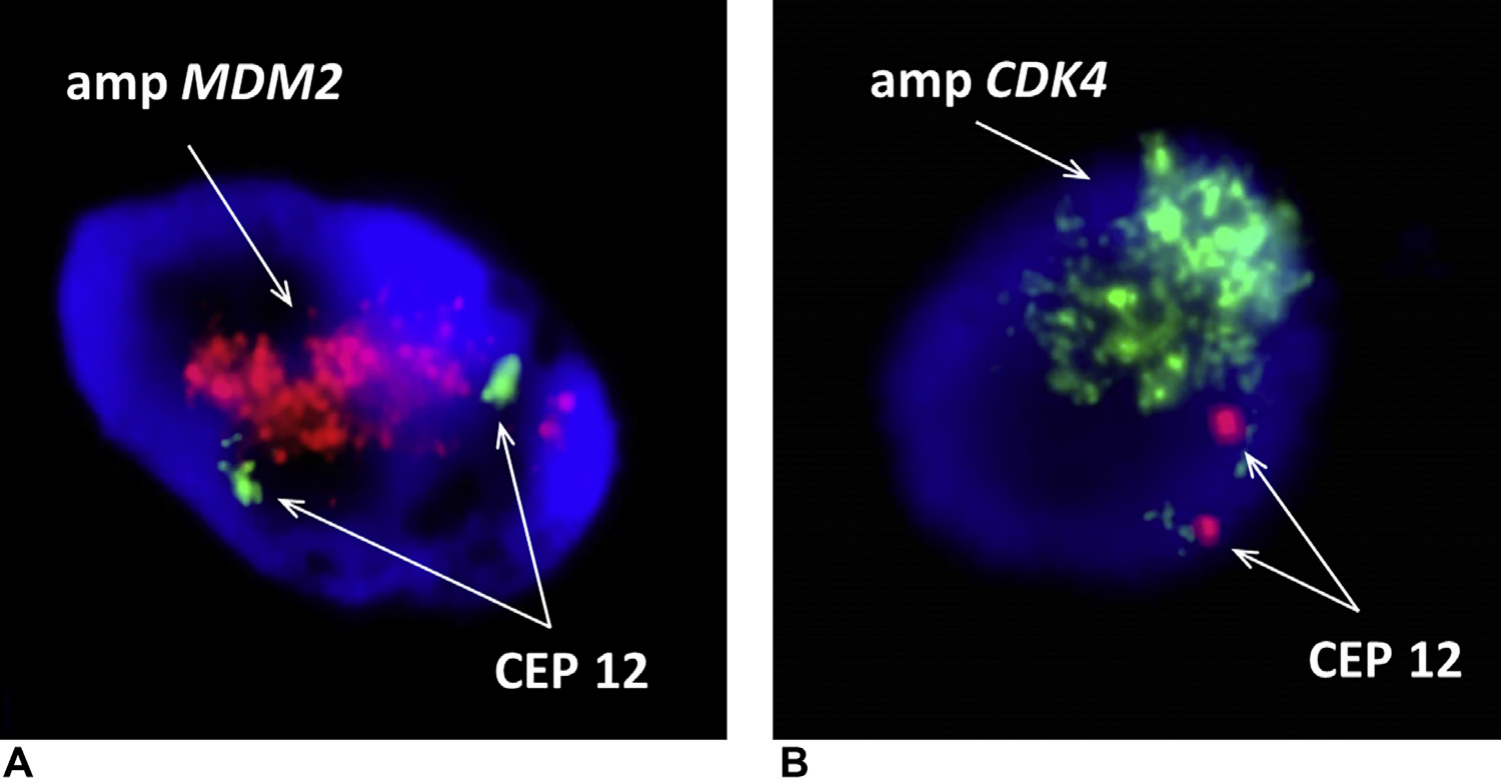
FISH analysis. Multiple clusters of signals showing amplification of *MDM2* (A) and *CDK4* (B) genes, respectively. Two centromeric signals (CEP 12) serve as a control of 2 copies of the chromosome 12.

**Fig. 12:**
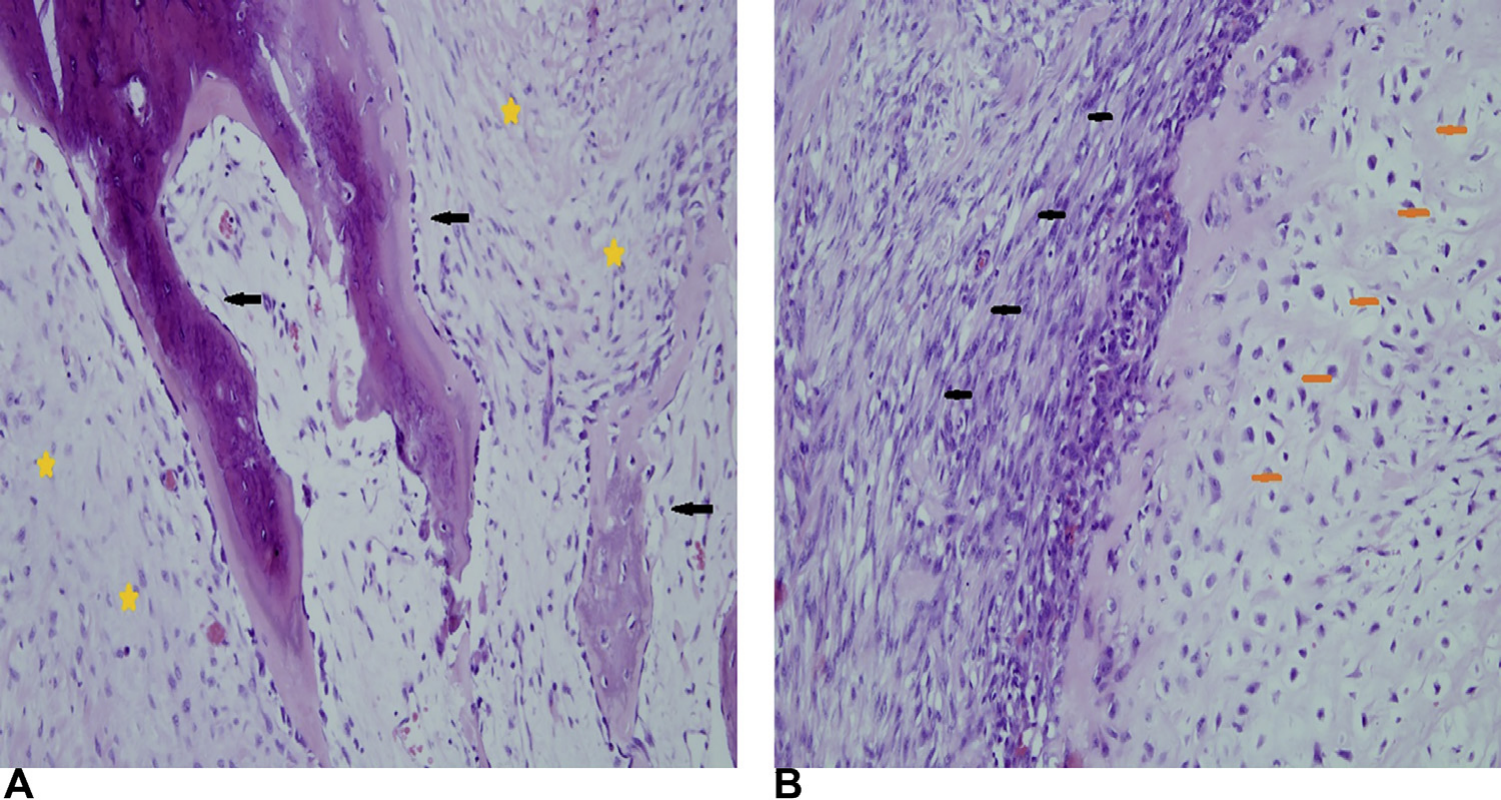
Biopsy of some areas of low-grade neoplasm with regularly arranged parallel, anastomosing bone trabeculae (arrows) separated by a hypocellular, sometimes more cellular spindle stroma (stars). There is a lack of cytologic atypia or increased mitotic activity (A); hyper-cellular, predominantly spindle cell proliferation with nuclear pleomorphism (black bars), extensive chondroid differentiation (yellow bars) (B); H&E, x100.

Fresh tissue biopsy cells were cultivated and used for genetic analysis. Cytogenetics showed complex clonal karyotype with the occurrence of ring chromosomes (Fig. 9). The array-based Comparative Genome Hybridization (aCGH) assay was performed to inspect the genetic abnormalities in the biopsy specimen. Despite the high degree of heterogeneity of tumor cells aCGH revealed the presence of amplified region of chromosome 12q13q15 (Fig. 10). In addition, some others CNV and aberrations have been detected, including additional amplifications on chromosome 12 and loss of chromosome Y (Supplementary Material). Fluorescent in situ hybridization (FISH) analysis was performed using locus specific probes for *MDM2* and *CDK4* genes in interphase cells of fresh tissue imprints and showed a high-level co-amplification of both genes in a 12q13-15 region (Fig. 11). Definitive histology showed presence of the pleomorphic spindle and oval cells with prominent mitotic activity and infiltrative growing into adjacent soft tissue (Figs. 8C and D). In addition to this pattern, there were some areas of low-grade neoplasm. This part of tumor consisted of regularly arranged parallel or anastomosing bony trabeculae separated by a hypocellular, sometimes intermediate cellular spindle stroma (Fig. 12A). These stromal spindle cells lack cytologic atypia or increased mitotic activity. There was an extensive chondroid differentiation (Fig. 12B). Further investigation by CT scan revealed metastases in the lungs.

Based on all these findings, the primary diagnosis was corrected to the focal dedifferentiated parosteal osteosarcoma with extensive cartilaginous differentiation. The patient underwent a leg amputation. In July 2017 passed away.

## Discussion

Despite the fact that parosteal osteosarcoma is the most common bone-surface tumor, it is often misdiagnosed as myositis ossificans. However, both diseases differ from each other in some distinct points.

While the parosteal osteosarcoma occurs most frequently in the third and fourth decade of life, myositis ossificans affects young patients, usually physically active males, in the second and third decades of life. Description of development stages of MO slightly varies between authors, usually 3 overlapping stages of evolution are commonly described: early, intermediate, and mature. The early stage occurs during the first 4 weeks following a musculoskeletal injury. It may begin with pain, erythema, swelling, fever, and modest decrease of joint movement. As the lesion matures through the intermediate stages (4-8 weeks), calcification becomes apparent radiographically. The mature stage follows, characterized by pronounced peripheral bone formation. By 5 to 6 months it develops into a painless, firm, well-demarcated ossified mass averaging 3 to 6 cm in the greater diameter. Lesion maturation continues during the following months, culminating in consolidation and, finally, regression [18,19].

On the other hand, POS develops over the months and years, rather than weeks. It primary affects the metaphyseal region of long bones and can reach enormous proportions if not treated. The tumor extends to the soft tissues and is connected to the adjacent bone. The mass feels hard to the touch. Patient in our case was 44-years old at the time of the first clinical manifestations. The first symptoms were swelling presented 3 years after he fell off his bike. Swelling had a slightly progressive nature.

On a roentgenogram, the parosteal osteosarcoma appears as an ossifying mass attached to the cortex with a broad base. The tumor tends to wrap around the involved bone as it grows. The underlying cortex can be normal, thickened, or destroyed [5]. In contrast, traumatic myositis ossificans has a characteristic “zoning” phenomenon usually seen by 4 weeks after the trauma. This phenomenon is characterized by a peripheral rim of calcification surrounding a central zone of lucency [20]. However, the mature lesions are often densely mineralized, making it more difficult to appreciate the zoning pattern of maturation [21].

CT of POS provides a fine determination of the tumor size, good visualization of radiolucent areas within the tumor and satellite lesions. Radiolucent area often represents high-grade sarcoma. The size of the lesion has a prognostic value. Identification of the satellite nodules provides valuable information for treatment as well. Patients who were treated by a limb-salvage procedure and these nodules were not removed entirely; the frequency of recurrent tumor growth would be higher [22]. The appearance of myositis ossificans on CT imaging varies with the age of the lesion. The CT image of the early MO shows an area of low attenuation indicating the soft-tissue portion of the mass. CT at 2 to 6 weeks can eventually reveal peripheral calcification. Maturity is shown by a diffuse, dense mineralization after 6 to 8 weeks [23].

Magnetic resonance imaging (MRI) is the most suitable method to evaluate medullary involvement in parosteal osteosarcomas. In contrast, MRI findings of myositis ossificans are considered nonspecific, but there is no medullary extension [23].

The scintigraphic findings of parosteal osteosarcoma can provide valuable insights that may aid in distinguishing it from its mimicker, osteochondroma. In parosteal osteosarcoma, the scintigraphy typically shows increased uptake across all 3 phases: vascular supply phase, blood pool phase, and delayed imaging. In contrast, osteochondroma shows only mild uptake on delayed imaging phase. Bone scintigraphy is a sensitive imaging modality for detection of the metastases, as it was in the case of presented patient.

The difficulty in establishing the primary diagnosis reflects the rareness of the lesion and the relative inexperience of referring surgeons, radiologists and pathologists [24]. Surface osteosarcomas can mimic benign conditions, such as myositis ossificans, osteochondroma, subperiosteal osteomyelitis, surface osteoma, and parosteal lipoma [5]. In contrast with POS, myositis ossificans is denser peripherally and is usually not attached to the cortex [25]. However, half of the cases, the ossification can go deeper and adhere to the periosteum. In these cases, the lesion is known as parosteal myositis ossificans [20]. In comparison with the osteochondroma, there is no usual continuity of the exterior of the mass with the adjacent cortex, and its inside is not in continuity with the underlying medullary cavity [26]. The key characteristics of parosteal osteosarcoma and its common mimickers are summarized in Table 1.

**Table 1:** This table summarizes the key characteristics of parosteal osteosarcoma and its common mimickers (osteochondroma, myositis ossificans, surface osteoma).

Imaging features	Osteochondroma	Myositis ossificans	Surface osteoma	Parosteal osteosarcoma
History of trauma	No	Yes	No	No
Development	Benign tumor	Weeks	Benign tumor	Months to years
Medullary contiguity	Yes	No	No	No
Cartilaginous cap	Yes	No	No	Rare, osteochondroma-like
Cortical destruction	No	No	No	Yes
Medullary invasion	No	No	No	Yes
Wraps around bone	No	No	No	Yes
Soft tissue mass	No	No	No	Yes
Surrounding soft tissue	Displacement	Displacement	Displacement	Invasion
Interface with soft tissue	Distinct interface	Distinct interface	Distinct interface	Indistinct interface
Cleavage plane	No	Yes, early on	No	Yes, two-thirds of the time
Lucencies within tumor	No	No	No	Yes
Zonal phenomenon (peripheral rim of calcification surrounding a central zone of lucency)	No	Yes	No	No
Scintigraphy				
Vascular supply phase	Normal	Normal	Normal	Increased uptake
Blood pool phase	Normal	Normal	Normal	Increased uptake

It compares various factors and diagnostic features, enabling differentiation between these conditions, suggesting the likely pathology, and aiding in diagnostic decision-making.

Histological reading of parosteal osteosarcoma is not the simplest task as the tumor does not have the typical hallmarks of malignancy. Therefore, many cases are considered to be benign, or reactive lesions, respectively. This might lead to inadequate surgical management [24]. Any lesions that are radiologically considered to be doubtful must be biopsied and histologically analyzed [5]. Histologically, 3 grades of parosteal osteosarcoma can be recognized (Grade 1–low; Grade 2–intermediated; Grade 3–dedifferentiated) with most frequent occurrence of the Grade 1/Grade 2 that either lack or shows only minimal anaplasia. That is one of the main sources of difficulties to recognize these entities as neoplasms on the cytological basis alone. Microscopic examination demonstrates parallel, well-formed bony trabeculae in a hypocellular stroma with or without osteoblastic rimming [5]. The initial histology findings were consistent with this description. Subsequent core-cut biopsy, performed in our institution, showed lack of typical pattern of low-grade, bone forming, hypo-cellular neoplasm. In a small specimen, there was observed cellular, predominantly spindle cell proliferation with nuclear pleomorphism and increased mitotic activity. The conclusion of this pilot biopsy was a high-grade spindle-cell sarcoma versus dedifferentiated MFH-like osteosarcoma. The growth pattern and continuity with the bone pointed out the possibility of dedifferentiation of formerly low-grade parosteal osteosarcoma. A bit clearer diagnosis was done from a definitive biopsy of the tumor after leg amputation. Although there were many areas of the tumor composed of hypercellular high-grade spindle cell proliferation, which was in accordance with the previous biopsy findings, the areas of low-grade bone forming osteosarcoma were observed as well. In addition, there were present some extensive areas of chondroid differentiation and necrotic areas.

On the other hand, histologic samples of myositis ossificans show 3 different zones: the central undifferentiated zone, the surrounding zone of the immature osteoid formation, and the peripheral zone with mature bone [20]. A biopsy specimen obtained after ossification shows primarily mature lamellar bone [20,21].

Common cytogenetic abnormality found in POS is a presence of supernumerary ring chromosomes. These chromosomes carry amplified material from chromosome 12 with minimal common region 12q13-q15 [15,27]. Cytogenetic analysis showed several ring chromosomes in the biopsy samples (Fig. 9). However, this approach is not able to reveal origin of their genetic material. This purpose suits better the array-based comparative genomic hybridization. The aCGH revealed amplification of region 12q13-q15 with genes *CDK4* and *MDM2*. Both of these genes play a key role in regulation of cell cycle and are hallmark of the POS [15,16]. Subsequent FISH confirmed amplification of both genes (Fig. 11). Thus, co-amplification of *CDK4/MDM2* and the presence of ring chromosomes supported even stronger diagnosis of parosteal osteosarcoma.

In addition, aCGH revealed some other aberrations that were not considered to be causal. These variants were not confirmed by any other method (Supplementary information).

Surgery remains the treatment of choice for osteosarcomas. However, nearly all tumors recur locally if not removed adequately. Wide excision with more than a 1 cm surgical margin is considered adequate to treat parosteal osteosarcoma at any surgical grade or stage [28]. In patients with advanced tumors containing pleomorphic areas and/or inadequate placed biopsies or previously taken insufficient surgical treatment, the amputation should be undertaken [29].

In our case, patient presented with a large tumor with medullary, soft tissue and neurovascular structures involved. Therefore, limb salvage procedure was not possible to recommend. Patient developed metastatic disease that showed signs of late stages of disease progression. Metastases are frequently found in the lungs, abdomen and some other rare locations [30]. If metastases appear, the prognosis is poor.

## Conclusions

Parosteal osteosarcoma is a rare low-grade malignant bone tumor. Because of its rareness, POS is often misinterpreted as the benign myositis ossificans or osteochondroma. Correct and timely diagnosis is a key assumption for efficient treatment. The treatment of choice is a surgical wide excision or amputation in the cases of recurrent disease, respectively. Presented case of patient with parosteal osteosarcoma is an example of how important is the cooperation of specialists coming from different fields, including orthopedic surgeons, radiologists, pathologists, and molecular geneticists.

## Author contributions

The authors contributed equally to the presented work.

## Patient consent

Written informed consent was obtained from patient prior to the interview.

## Appendix Supplementary materials

Image, application 1
